# Virology, Epidemiology, Pathogenesis, and Control of COVID-19

**DOI:** 10.3390/v12040372

**Published:** 2020-03-27

**Authors:** Yuefei Jin, Haiyan Yang, Wangquan Ji, Weidong Wu, Shuaiyin Chen, Weiguo Zhang, Guangcai Duan

**Affiliations:** 1 Department of Epidemiology, College of Public Health, Zhengzhou University, Zhengzhou 450001, China; jyf201907@zzu.edu.cn (Y.J.); yhy@zzu.edu.cn (H.Y.); jwq1995zzu@163.com (W.J.); sychen@zzu.edu.cn (S.C.); wzhang033@icloud.com (W.Z.); 2 School of Public Health, Xinxiang Medical University, Xinxiang 453003, China; wdwu2013@126.com; 3 Department of Immunology, Duke University Medical Center, Durham, NC 27710, USA

**Keywords:** SARS-CoV-2, COVID-19, epidemiology, pathogenesis, therapeutics

## Abstract

The outbreak of emerging severe acute respiratory syndrome coronavirus 2 (SARS-CoV-2) disease (COVID-19) in China has been brought to global attention and declared a pandemic by the World Health Organization (WHO) on March 11, 2020. Scientific advancements since the pandemic of severe acute respiratory syndrome (SARS) in 2002~2003 and Middle East respiratory syndrome (MERS) in 2012 have accelerated our understanding of the epidemiology and pathogenesis of SARS-CoV-2 and the development of therapeutics to treat viral infection. As no specific therapeutics and vaccines are available for disease control, the epidemic of COVID-19 is posing a great threat for global public health. To provide a comprehensive summary to public health authorities and potential readers worldwide, we detail the present understanding of COVID-19 and introduce the current state of development of measures in this review.

## 1. Introduction

At the end of 2019, a cluster of pneumonia patients with an unidentified cause emerged in Wuhan, Hubei Province, China [1]. Since then, outbreaks and sporadic human infections have resulted in more than 80,000 laboratory confirmed cases (update on March 23, 2020) across mainland China. Through the analysis of sequence, this unidentified pneumonia was considered to be caused by a novel coronavirus (CoV) named 2019-nCoV [2]. Subsequently, the World Health Organization (WHO) announced a standard format of Coronavirus Disease-2019 (COVID-19), according to its nomenclature, for this novel coronavirus pneumonia on February 11, 2020 [3]. On the same day, the International Committee on Taxonomy of Viruses (ICTV) named this novel coronavirus as SARS-CoV-2 [4]. So far, the SARS-CoV-2 infection is still spreading, and this virus poses a serious threat to public health, though joint prevention and quarantine mechanisms in almost all provinces of mainland China have been confirmed to be enacted. Due to a lack of specific antiviral treatments and pressure of clinical treatment, thousands of severe cases have died every day worldwide. In this review, we discuss the virology, clinical and molecular epidemiology, diagnosis, pathogenesis, and potential therapeutics for treatment of this infection.

## 2. Virology

### 2.1. Origin, Classification, and Genome

**Figure 1:**
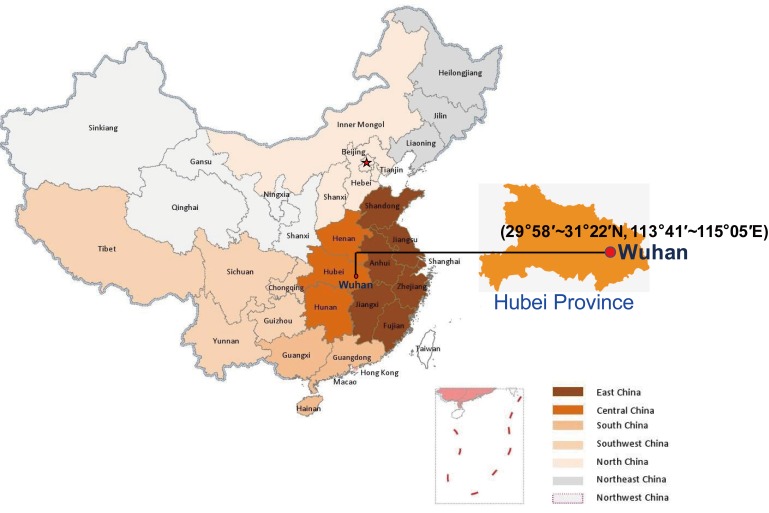
Geographic location of Wuhan, Hubei Province in China. Hubei Province is located in the central area of China, and the provincial capital is Wuhan.

At the end of 2019, COVID-19 emerged in several local hospitals of Wuhan, Hubei Province, China (Figure 1). Based on clinical manifestations, blood tests, and chest radiographs, this disease was diagnosed as virus-induced pneumonia by clinicians. Initial epidemiological investigation suggested that a majority of suspected cases were associated with their presence (exposure) in a local Huanan seafood market. Notably, not just seafood, but many kinds of live wild animals were available for sale in this market all year round before it was forced to close on January 1, 2020. As expected, SARS-CoV-2 was isolated in environmental samples of the Huanan Seafood Market by China Center for Disease Control and Prevention (CDC), implying the origin of the outbreak. However, such a decisive conclusion was disputed because the earliest case had had no reported link connection to the mentioned market [5]. In addition, it was found that at least two different strains of SARS-CoV-2 had occurred a few months earlier before COVID-19 was officially reported [6]. A recently phyloepidemiologic analysis suggests that SARS-CoV-2 at the Huanan Seafood Market could have been imported from other places [7]. To date, it remains inconsistent with regard to the origin of SARS-CoV-2, and epidemiologic and etiologic investigations are being conducted by Chinese health authorities.

SARS-CoV-2 was first isolated in the bronchoalveolar lavage fluid (BALF) of three COVID-19 patients from Wuhan Jinyintan Hospital on December 30, 2019 [2]. After sequence and evolutionary tree analysis, SARS-CoV-2 was considered as a member of β-CoVs [2,8]. The CoVs family is a class of enveloped, positive-sense single-stranded RNA viruses having an extensive range of natural roots. These viruses can cause respiratory, enteric, hepatic, and neurologic diseases [9,10]. The CoVs are genotypically and serologically divided into four subfamilies: α, β, γ, and δ-CoVs. Human CoV infections are caused by α- and β-CoVs [9,10]. SARS coronavirus (SARS-CoV) and MERS coronavirus (MERS-CoV) are members of β-CoVs [9]. Genome-wide phylogenetic analysis indicates that SARS-CoV-2 shares 79.5% and 50% sequence identity to SARS-CoV and MERS-CoV, respectively [2,8,11]. However, there is 94.6% sequence identity between the seven conserved replicase domains in ORF1ab of SARS-CoV-2 and SARS-CoV [8], and less than 90% sequence identity between those of SARS-CoV-2 and other β-CoVs [2], implying that SARS-CoV-2 belongs to the lineage B (Sarbecovirus) of β-CoVs [12].

**Figure 2:**
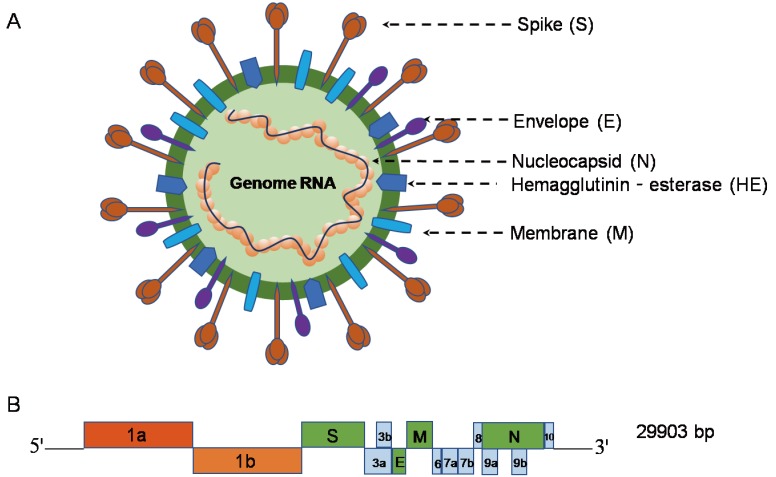
β-coronavirus particle and genome [9] (**A**) The β-coronavirus particle. β-coronavirus is an enveloped, nonsegmented, positive-sense single-stranded RNA virus genome in a size ranging from 29.9 kb. The virion has a nucleocapsid composed of genomic RNA and phosphorylated nucleocapsid (N) protein, which is buried inside phospholipid bilayers and covered by the spike glycoprotein trimmer (S). The membrane (M) protein hemagglutinin-esterase (HE) and the envelope (E) protein are located among the S proteins in the virus envelope. (**B**) 5′ and 3′ terminal sequences of the SARS-CoV-2 genome. The gene order is 5′-replicase ORF1ab-S-envelope(E)-membrane(M)-N-3′. ORF3ab, ORF6, ORF7ab, ORF8, ORF9ab, and ORF10 are located at the predicted positions shown in the picture. 1a, 1b, 3a, 3b, 6, 7a, 7b, 8, 9a, 9b, 10 in the picture represent different ORF genes.

As shown in Figure 2A, similar to other β-CoVs, the SARS-CoV-2 virion with a genome size of 29.9 kb [13] possesses a nucleocapsid composed of genomic RNA and phosphorylated nucleocapsid (N) protein. The nucleocapsid is buried inside phospholipid bilayers and covered by two different types of spike proteins: the spike glycoprotein trimmer (S) that exists in all CoVs, and the hemagglutinin-esterase (HE) only shared among some CoVs. The membrane (M) protein and the envelope (E) protein are located among the S proteins in the viral envelope [12]. The SARS-CoV-2 genome has 5′ and 3′ terminal sequences (265 nt at the 5′ terminal and 229 nt at the 3′ terminal region), which is typical of β-CoVs, with a gene order 5′-replicase open reading frame (ORF) 1ab-S-envelope(E)-membrane(M)-N-3′ (Figure 2B). The predicted S, ORF3a, E, M, and N genes of SARS-CoV-2 are 3822, 828, 228, 669, and 1260 nt in length, respectively. Similar to SARS-CoV, SARS-CoV-2 carries a predicted ORF8 gene (366 nt in length) located between the M and N ORF genes [12].

### 2.2. Physicochemical Properties

The virus particle has a diameter of 60~100 nm and appears round or oval [14]. Most of the knowledge about the physicochemical properties of CoVs comes from SARS-CoV and MERS-CoV. SARS-CoV-2 can be inactivated by UV or heated at 56 °C 30 min, and also sensitive to most disinfectants such as diethyl ether, 75% ethanol, chlorine, peracetic acid, and chloroform [14]. It has been reported that SARS-CoV-2 was more stable on plastic and stainless steel than on copper and cardboard, and viable virus was detected up to 72 h after application to these surfaces. On cardboard, the half-life of SARS-CoV-2 was longer than that of SARS-CoV and the longest viability of both viruses was on stainless steel and plastic [15].

### 2.3. Receptor Interactions and Cell Entry

Human angiotensin-converting enzyme 2 (ACE2) is a functional receptor hijacked by SARS-CoV-2 for cell entry, similar to SARS-CoV [8,16]. ACE2 is a type I membrane protein expressed in lung, heart, kidney, and intestine mainly associated with cardiovascular diseases [17]. The full-length ACE2 consists of an N-terminal peptidase domain (PD) and a C terminal Collectrin-like domain (CLD) that ends with a single transmembrane helix and an~40-residue intracellular segment [17]. In addition to cleavage of angiotensin (Ang) I to produce Ang-(1-9), ACE2 also provides a direct binding site for the S proteins of CoVs [17]. The S protein of CoVs exists in a metastable pre-fusion conformation that undergoes a dramatic structural rearrangement to fuse the viral membrane with the host cell membrane [10]. This process is triggered by the S1 subunit and a host–cell receptor binding, which destabilizes the pre-fusion trimer, resulting in the S1 subunit shedding and the S2 subunit transition to a highly stable post-fusion conformation [10]. To engage a host–cell receptor, the receptor-binding domain (RBD) of S1 undergoes hinge-like conformational movements that transiently hide or expose the determinants of receptor binding [18]. In order to figure out the potential of SARS-CoV-2 to infect humans, the receptor-binding domain (RBD) of its S protein, which is in contact with ACE2, was analyzed. The biophysical and structural evidence suggests that SARS-CoV-2 S protein likely binds to human ACE2 with 10–20 fold higher affinity than SARS-CoV [19]. Another structural evidence suggests that the ACE2-B0AT1 complex can bind two S proteins simultaneously [20].

### 2.4. Evolutionary Insights into the Ecology of SARS-CoV-2

All human CoVs may be of zoonotic origin, and bats are most likely the natural hosts for all presently known CoVs [21]. During the SARS pandemic in 2002 and 2003, the first hints pointed to a zoonotic origin of the SARS-CoV, with civets as the suspected natural source of human infection [22]. Genetically diversified SARS-like CoVs were then found in Chinese Rhinolophid bats, and two novel bat CoVs from Chinese horseshoe bats (family: Rhinolophidae) in Yunnan Province, China are reported to be very closely related to SARS-CoV, implying that Chinese horseshoe bats are natural host of SARS-CoV [23,24]. Regarding SARS-CoV-2, it showed a high sequence identity to some bat CoVs such as BatCoV RaTG13 (96% nt identity to SARS-CoV-2) previously detected in Rhinolophusaffinis from Yunnan Province, indicating a bat origin of SARS-CoV-2 [12,23].

**Figure 3:**
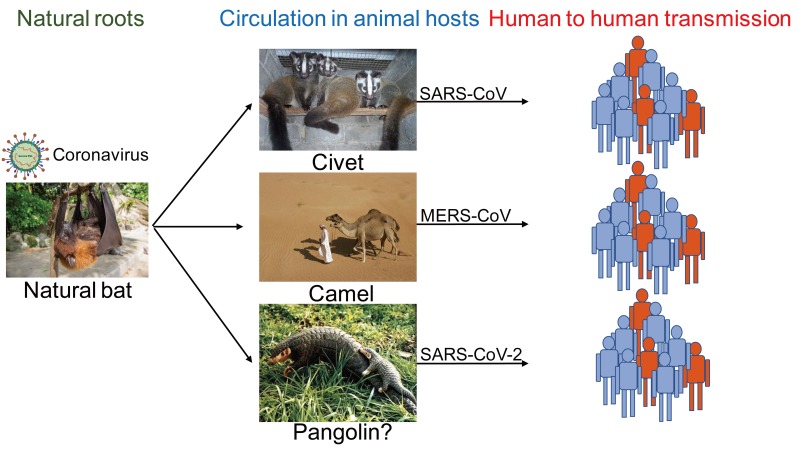
Ecology of emerging coronaviruses SARS-CoV, MERS-CoV, and SARS-CoV-2 are all bat origin coronaviruses, which cause human infections after circulation in animal hosts of civet, camel, and pangolin.

Generally, bat habitats are far from human activity areas, and the virus was probably transmitted to humans by another animal host. Bat SARS like-CoVs cannot directly infect humans unless they undergo mutation or recombination in animal hosts [22]. For example, animal hosts of SARS-CoV and MERS-CoV are the civet and camel (Figure 3) before transmission to humans. Regarding the intermediate animal host of SARS-CoV-2, it has been reported that the sequence identity between pangolin origin CoVs and SARS-CoV-2 is 99%, indicating that SARS-CoV-2 may be of pangolin origin [25]. Many studies in China are tracking other potential animal hosts of SARS-CoV-2, which is of great significance for the prevention and control of COVID-19.

### 2.5. Genomic Variation

The initial 10 genomic sequences of SARS-CoV-2 obtained from the nine COVID-19 patients were extremely similar, exhibiting more than 99.98% sequence identity, implying that not much variation has taken place [8,11]. A recent study indicates that 120 substitution sites were evenly distributed in eight coding regions, without evident recombination events [7]. However, Tang et al. found that SARS-CoV-2 had evolved into two major types of *L* and *S*, based on analyses of 103 genomes. Due to severe selective pressure on the *L* type, the *L* type might be more aggressive and spread more quickly, while the *S* type might remain milder due to relatively weaker selective pressure [26]. Due to the unstable nature of RNA viruses, the continuous surveillance of SARS-CoV-2 from humans or animals is extremely important for disease control.

## 3. Epidemiology

### 3.1. Source of Infection

Currently, COVID-19 patients are the main source of infection, and severe patients are considered to be more contagious than mild ones. Asymptomatically infected persons or patients in incubation who show no signs or symptoms of respiratory infection proven to shed infectious virus, may also be potential sources of infection [27]. Additionally, samples taken from patients recovered from COVID-19 continuously show a positive RT-PCR test [28], which has never been seen in the history of human infectious diseases. In other words, asymptomatically infected persons and patients in incubation or recovered from COVID-19 may pose serious challenges for disease prevention and control.

### 3.2. Spectrum of Infection

COVID-19 has been considered as a type of self-limiting infectious disease, and most cases with mild symptoms can recover in 1–2 weeks. SARS-CoV-2 infection can cause five different outcomes: asymptomatically infected persons (1.2%); mild to medium cases (80.9%); severe cases (13.8%); critical case (4.7%); and death (2.3% in all reported cases) [29]. The latest study indicates that the proportion of asymptomatic infection in children under 10-years old is as high as 15.8% [30]. Therefore, the proportion of asymptomatic infection should be further uncovered in the future.

### 3.3. Clinical Features

In the initial 41 patients [5], fever (98%), cough (76%), and myalgia or fatigue (44%) were the most common symptoms. Less common symptoms were sputum production (28%), headache (8%), hemoptysis (5%), and diarrhea (3%). More than half of patients developed dyspnea. The average incubation period and basic reproduction number (R0) were estimated to be 5.2 d (95% CI: 4.1–7.0) and 2.2 (95% CI, 1.4–3.9), respectively [1,29]. Blood test showed normal or reduced (25%) white blood cell count and lymphopenia (65%) [5]. A total of 98% of patients had bilateral involvement under chest CT. Typical findings of chest CT images of ICU patients on admission were bilateral multiple lobular and subsegmental areas of consolidation. The representative chest CT findings of non-ICU patients showed bilateral ground-glass opacity and subsegmental areas of consolidation [2,5]. Analysis of 1324 laboratory confirmed cases showed that fever (87.9%) and cough (67.7%) were still the most common symptoms, while diarrhea is uncommon. Lymphopenia was observed in 82.1% of patients admitted to ICU [31].

### 3.4. Epidemiological Characteristics in Mainland China

**Figure 4:**
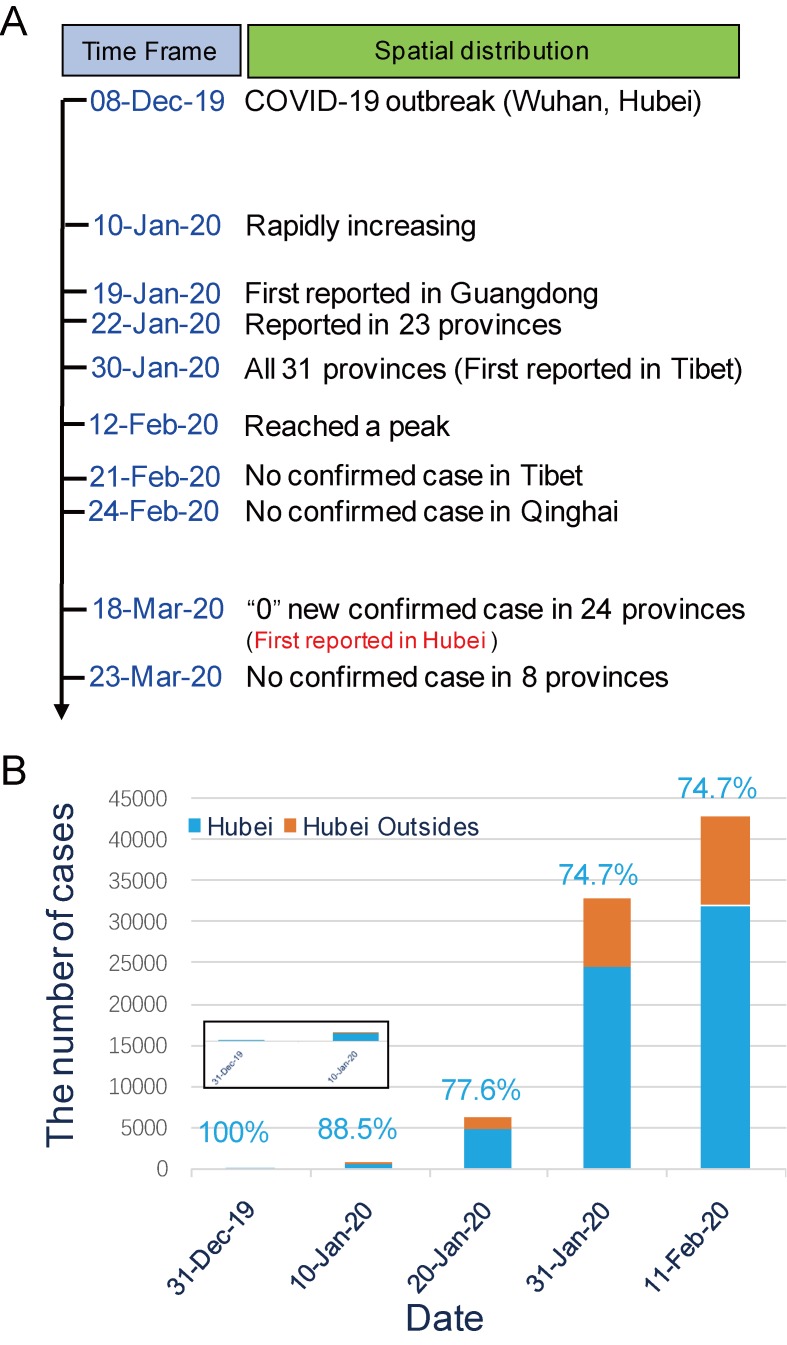
Spatiotemporal distribution of COVID-19. (**A**) The spread and decline of COVID-19 in mainland China over time. The time point (red words) of “0” new confirmed case first reported in Hubei Province was 18 March, 2020. (**B**) Distribution of cases with different onset times before 11 February 2020.

Spatiotemporal distribution. The initial outbreak (8 December 2020) only occurred in Wuhan and its surroundings inHubei Province before an imported case was first reported in Guangdong Province on January 19, 2020 [1,29]. As of January 30, 2020, when the first imported case in Tibet Province was reported, COVID-19 had spread to all 31 provinces in mainland China (Figure 4A). Until 11 February2020, 44,672 cases were reported in all 31 provinces of mainland China (74.7% in Hubei). Among them, 0.2% (100% in Hubei), 1.7% (88.5% in Hubei), 13.8% (77.6% in Hubei), and 73.1% (74.7% in Hubei) of cases were onset before 31 December 2019, 10 January 2020, 20 January 2020, and 31 January 2020, respectively (Figure 4B). The number of reported cases rose rapidly after 10 January 2020, and reached a peak on 12 February 2020 [32]. Through the analysis of 1688 healthcare confirmed cases with severe symptoms, there were 1080 cases in Wuhan, accounting for 64.0% of the total incidence, 394 cases (23.3%) in Hubei except Wuhan, and 214 cases (12.7%) nationwide, except for Hubei [29]. Tibet and Qinghai Provinces have had no confirmed cases since 21 February 2020 and 24 February 2020, respectively (Figure 4A) [33]. On 18 March 2020, “0” new confirmed cases was first reported in Hubei Province, and a total of 24 provinces in mainland China had consecutively reported “0” new confirmed cases. Until 23 March 2020, 81,773 cases (427 imported cases from abroad) were cumulative reported in 31 provinces of mainland China, and the number of new confirmed cases have mainly come from abroad and eight provinces have had no confirmed cases (Figure 4A) [34].

Population distribution. China CDC data [29] showed that patients were mainly concentrated at the age of 30–79, accounting for 89.8%, 88.6%, and 86.6% of confirmed cases in Wuhan, Hubei, and mainland China, respectively. The gender ratio (male/female) of confirmed cases in Wuhan, Hubei, and mainland China was 0.99:1, 1.04:1, and 1.06:1, respectively. The proportion of infected healthcare workers and farmers was 2.09% and 22%, respectively [29].

Case-fatality rate. The total case-fatality rate was 2.3% of 44,672 confirmed cases, while the total case-fatality rate in Hubei and its surroundings was 2.9% and 0.4%, respectively [29]. In contrast, the total case-fatality rate of SARS and MERS was 9.6% [35] and 34%, respectively [36]. In all COVID-19 patients over 80-years old, the case-fatality rate wasas high as 14.8%. The case-fatality rate of males and femaleswas2.8% and 1.7%, respectively [29]. Patients with underlying basic disorders showed poor prognosis. The case-fatality rate of cases without basic disorders was as low as 0.9%, while the case-fatality rate of cases with cardiovascular disease, diabetes, chronic respiratory disease, hypertension, and cancer was10.5%, 7.3%, 6.3%, 6.0%, and 5.6%, respectively [29]. Notably, critical cases had the highest case-fatality rate of 49% [29]. As for healthcare workers, the case-fatality rate was approximately 0.17% of 3019 cases [29].

### 3.5. Other Regions

According to the WHO data updated on March 23, 2020 [37], 190countriesor areas have reported 332,218 laboratory confirmed cases including 14,510 deaths. The total case-fatality rate of global cases outside China is 4.5%. More attention should be paid to Italy, Spain, the USA, Germany, France, and Iran with more severe outbreaks [37]. The top five countries with the highest cumulative confirmed cases in the world are China (24.6%), Italy (17.8%), USA (9.5%), Spain (8.6%), and Germany (7.5%). Higher case-fatality rates were found in Italy (9.3%), Iran (7.8%), and Spain (6.0%) [37].

### 3.6. Routes of Transmission

Currently, respiratory droplets and contact transmission are considered to be the main transmission routes. Recent reports indicate that SARS-CoV-2 can be detected in the urine and stool of laboratory confirmed patients, implying a risk of fecal–oral transmission [14]. However, it is not yet certain that the consumption of virus-contaminated foods will cause infection and transmission. There is still no evidence that SARS-CoV-2 can be transmitted through aerosols or from mother to baby during pregnancy or childbirth.

### 3.7. Herd Susceptibility

As an emerging infectious disease, the population of all races and ages is generally susceptible. In mainland China, 30~65-year-old persons account for 71.45% and children under 10-years-old account for 0.35% [31]. Elderly people and persons with underlying basic disorders such as asthma, diabetes, cardiovascular diseases, and cancer may be more susceptible to SARS-CoV-2 [38,39]. Smoking and obesity are also susceptible factors [40,41].

### 3.8. High-Risk Population

Persons who are in close contact with patients or subclinically symptomatic infected persons are part of the high-risk population. High infection risk is also considered in healthcare workers and the family members of patients [42].

## 4. Diagnosis

### 4.1. Nucleic Acid Test

Viral diagnostics is one important part of our armamentarium against COVID-19. After initial outbreak, diagnostic tests based on the detection of the viral sequence by RT-PCR or next generation sequencing platforms soon became available. Subsequently, many biotechnology companies have successfully developed nucleic acid detection kits, and the China Food and Drug Administration (CFDA) has urgently approved a batch of fluorescent quantitative kits and sequencing systems [43]. The main concern related to the nucleic acid test is false negatives. To solve the problem of low detection efficiency, some improved rapid viral nucleic acid diagnostic tests have been invented. Particularly, a nucleic acid test paper, which can be used for the rapid detection of SARS-CoV-2 with the naked eye observation within three minutes, has been successfully developed [44].

### 4.2. Serologic Diagnosis

It has been shown that patients with SARS-CoV-2 infection possess acute serological responses [8]. Combined with immunochromatography, colloidal gold, and other technologies, relevant detection reagents have been developed rapidly [45,46].

### 4.3. CRISPR/Cas13 System

The Cas13-based SHERLOCK (specific high-sensitivity enzymatic reporter unlocking) platform has been widely used to detect Zika virus (ZIKV) and dengue virus (DENV) in patient samples at concentrations as low as 1 copy per microliter [47]. Recently, Zhang et al. released a CRISPR/Cas13-based SHERLOCK technology to detect SARS-CoV-2. However, this CRISPR/Cas13 system remains to be verified because it has not been tested on clinical samples from COVID-19 patients.

### 4.4. Imaging Technology

Chest radiograph or CT is an important tool for COVID-19 diagnosis in clinical practice. The majority of COVID-19 cases have similar features on CT images including bilateral distribution of patchy shadows and ground glass opacity [48]. The great value of using the deep learning machine to extract radiological graphical features for COVID-19 diagnosis has been introduced [49]. Artificial Intelligence (AI) can accurately interpret the CT images of the suspected cases of the new crown within 20 s, and the accuracy rate of the analysis results reached 96%, greatly improving the diagnostic efficiency. This technique is already being used in clinical practice.

## 5. Pathogenesis

### 5.1. Virus Entry and Spread

**Figure 5:**
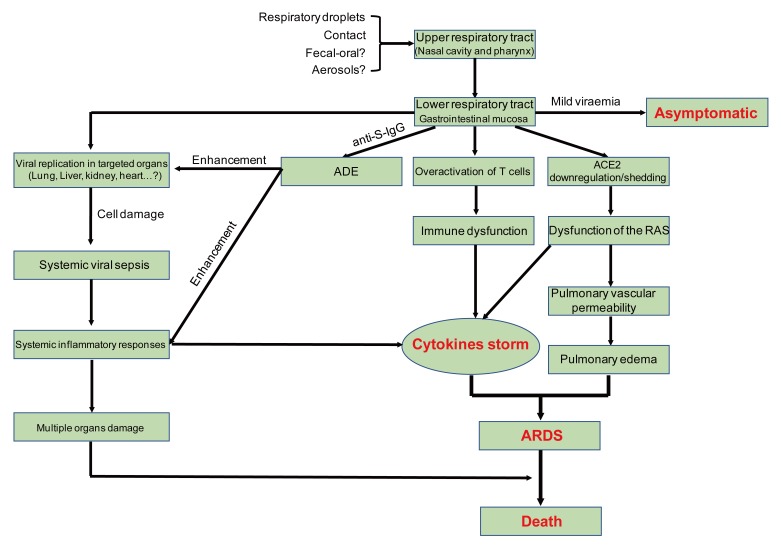
Postulated pathogenesis of SARS-CoV-2 infection. Antibody-dependent enhancement (ADE); ACE2: angiotensin-converting enzyme 2; RAS: renin-angiotensin system; ARDS: acute respiratory distress syndrome. Red words represent the important turning points in SARS-CoV-2 infection.

SARS-CoV-2 is transmitted predominantly via respiratory droplet, contact, and potential in fecal-oral [14]. Primary viral replication is presumed to occur in mucosal epithelium of upper respiratory tract (nasal cavity and pharynx), with further multiplication in lower respiratory tract and gastrointestinal mucosa [50], giving rise to a mild viremia. Few infections are controlled at this point and remain asymptomatic. Some patients have also exhibited non-respiratory symptoms such as acute liver and heart injury, kidney failure, diarrhea [5,51,52,53], implying multiple organ involvement. ACE2 is broadly expressed in nasal mucosa, bronchus, lung, heart, esophagus, kidney, stomach, bladder, and ileum, and these human organs are all vulnerable to SARS-CoV-2 [40]. Recently, potential pathogenicity of the SARS-CoV-2 to testicular tissues hasalso been proposed by clinicians, implying fertility concerns in young patients [54]. The postulated pathogenesis of SARS-CoV-2 infection is graphed in Figure 5.

### 5.2. Pathological Findings

The first report [55] of pathological findings from a severe COVID-19 showed pulmonary bilateral diffuse alveolar damage with cellular fibromyxoid exudates. The right lung showed evident desquamation of pneumocytes and hyaline membrane formation, indicating acute respiratory distress syndrome. The left lung tissue displayed pulmonary edema with hyaline membrane formation, suggestive of early-phase acute respiratory distress syndrome (ARDS). Interstitial mononuclear inflammatory infiltrates, dominated by lymphocytes, could be observed in both lungs. Multinucleated syncytial cells with atypical enlarged pneumocytes characterized by large nuclei, amphophilic granular cytoplasm, and prominent nucleoli were identified in the intra-alveolar spaces, indicating viral cytopathic-like changes. These pulmonary pathological findings extremely resemble those seen in SARS [56] and MERS [57]. Moderate microvascular steatosis and mild lobular and portal activity were observed in liver biopsy specimens, which might be caused by either SARS-CoV-2 infection or drug use. In addition, only a few interstitial mononuclear inflammatory infiltrates were found in the heart tissue, which means that SARS-CoV-2 might not directly impair the heart [55]. Massive mucus secretion in both lungs was found in death cases with COVID-19, which was different from SARS and MERS [58].

### 5.3. Acute Respiratory Distress Syndrome (ARDS)

ARDS is a life-threatening lung condition that prevents enough oxygen from getting to the lungs and into the circulation, accounting for mortality of most respiratory disorders and acute lung injury [59]. In fatal cases of human SARS-CoV, MERS-CoV, and SARS-CoV-2 infections, individuals exhibit severe respiratory distress requiring mechanical ventilation, and the histopathology findings also support ARDS [55,56,57]. Previous studies have found that genetic susceptibility, and inflammatory cytokines were closely related to the occurrence of ARDS. More than 40 candidate genes including ACE2, interleukin 10 (IL-10), tumor necrosis factor (TNF), and vascular endothelial growth factor (VEGF) among others have been considered to be associated with the development or outcome of ARDS [60]. Increased levels of plasma IL-6 and IL-8 were also demonstrated to be related to adverse outcomes of ARDS [59]. The above biomarkers suggest both a molecular explanation for the severe ARDS and a possible treatment for ARDS followingSARS-CoV-2 infection.

### 5.4. Cytokine Storm

Clinical findings showed exuberant inflammatory responses during SARS-CoV-2 infection, further resulting in uncontrolled pulmonary inflammation, likely a leading cause of case fatality. Rapid viral replication and cellular damage, virus-induced ACE2 downregulation and shedding, and antibody dependent enhancement (ADE) are responsible for aggressive inflammation caused by SARS-CoV-2, as concluded in a recently published review article [61]. SARS-CoV-2 hijacks the same entry receptor, ACE2, as SARS-CoV for infection, suggesting the likelihood of the same population of cells being targeted and infected [62]. The initial onset of rapid viral replication may cause massive epithelial and endothelial cell death and vascular leakage, triggering the production of exuberant pro-inflammatory cytokines and chemokines [63]. Loss of pulmonary ACE2 function has been proposed to be related to acute lung injury [64] because ACE2 downregulation and shedding can lead to dysfunction of the renin-angiotensin system (RAS), and further enhance inflammation and cause vascular permeability. For SARS-CoV, one confusing issueis that only a few patients, particularly those who produce neutralizing antibodies early, experience persistent inflammation, ARDS, and even sudden death, while most patients survive the inflammatory responses and clear the virus [61]. The above phenomenon also exists in SARS-CoV-2 infection. A possible underlying mechanism of antibody-dependent enhancement (ADE) has been proposed recently [61]. ADE, a well-known virology phenomenon, has been confirmed in multiple viral infections [65]. ADE can promote viral cellular uptake of infectious virus–antibody complexes following their interaction with Fc receptors (FcR), FcγR, or other receptors, resulting in enhanced infection of target cells [65]. The interaction of FcγR with the virus-anti-*S* protein-neutralizing antibodies (anti-*S*-IgG) complex may facilitate both inflammatory responses and persistent viral replication in the lungs of patients [61].

### 5.5. Immune Dysfunction

Peripheral CD4 and CD8 T cells showed reduction and hyperactivation in a severe patient. High concentrations of proinflammatory CD4 T cells and cytotoxic granules CD8 T cells were also determined, suggesting antiviral immune responses and overactivation of T cells [55]. Additionally, several studies have reported that lymphopenia is a common feature of COVID-19 [2,5], suggestive of a critical factor accounting for severity and mortality.

## 6. Potential Therapeutics

Currently, there are no specificantiviral drugs or vaccines for the control of SARS-CoV-2. Symptomatic treatment strategies are recommended for clinical practice [14]. Here, we summarize potential therapeutics available for the treatment of SARS-CoV-2.

### 6.1. Type I IFNs

Type I IFNs are antiviral cytokines that induce a large range of proteins that can impair viral replication in targeted cells. Previous studies have reported that IFN-β was superior against SARS-CoV compared to IFN-α [66]. Synergistic effects of leukocytic IFN-α with ribavirin [67] and IFN-β with ribavirin [68] against SARS-CoV were demonstrated in vitro.

### 6.2. Potential Antiviral Compounds

Ribavirin. During the outbreak of SARS in Hong Kong, ribavirin was broadly used for patients with or without concomitant use of steroids [69]. Ribavirin and IFN-β could synergistically inhibit SARS-associated CoV replication in vitro [68]. Due to adverse reactions, the proper dose of ribavirin in clinical application should be given carefully.

Lopinavir/ritonavir. The combination of lopinavir/ritonavir is widely used in the treatment of HIV infection. It has been reported that the use of lopinavir/ritonavir with ribavirin has a good therapeutic effect in SARS [70] and MERS [71]. Lopinavir/ritonavir has been recommended for clinical treatment for COVID-19.

Remdesivir. Remdesivir (RDV) was previously reported to restrain SARS-CoV in vivo [72], and the antiviral protection of RDV and IFN-β was found to be superior to that of lopinavir/ritonavir-IFN-β against MERS-CoV in vitro and in vivo. In addition, remdesivir was used in the treatment of the first COVID-19 patient in the United States [27] and was shown to have antiviral activity against SARS-CoV-2 in vitro [73]. However, its effectiveness and safety have not been verified in clinical trials yet.

Nelfinavir. Nelfinavir is a selective inhibitor of HIV protease, which has been shown to have a strong inhibition of SARS-CoV [74] implying a possible therapeutic for COVID-19.

Arbidol. Arbidol, a broad-spectrum antiviral compound, is able to block viral fusion against influenza viruses. In addition, arbidol and its derivative, arbidolmesylate, have been reported to have antiviral activity against SARS-CoV in vitro [75]. The antiviral activity of arbidol against SARS-CoV-2 has been confirmed in vitro and recommended for clinical treatment [14].

Chloroquine. Chloroquine has many interesting biochemical properties including antiviral effect [76]. It has been found to be a potent inhibitor of SARS-CoV through interfering with ACE2 [74]. Chloroquine can effectively inhibit SARS-CoV-2 in vitro [73], and is recommended for the clinical control of viral replication [14].

### 6.3. Convalescent Plasma

Recently, convalescent plasma has been widely recommended to be used for COVID-19 [77], but the effect of convalescent plasma cannot be discerned from the effects of patient comorbidities, stage of illness, or effect of other treatments.

### 6.4. Protective Monoclonal Antibody

It has been reported that the monoclonal antibody (mAb) can efficiently neutralize SARS-CoV and inhibit syncytia formation between cells expressing the *S* protein and those expressing the SARS-CoV receptor ACE2 [78]. However, mAbs can only recognize a single epitope, and the anti-infective effect may be limited. In addition, the development of mAbs requires a certain period of time, which is difficult to achieve in clinical application in a short time.

### 6.5. Others

Based on the virology of SARS-CoV-2, blocking the binding of *S* protein to ACE2 is important for the treatment of virus infection. ACE2 is an important component of the renin-angiotensin system (RAS). RAS inhibitors, ACEI and AT1R, may be potential therapeutic tools for COVID-19. Additionally, intravenous transplantation of ACE2-mesenchymal stem cells (MSCs), blocking of FcR with immunoglobulin (IVIG), and systemic anti-inflammatory drugs to reduce cytokine storm are also potential therapeutic strategies for severe COVID-19 [61,79]. Traditional Chinese medicines have also been found to have potential anti-SARS-CoV-2 activity [80].

## 7. Vaccine Development

Vaccination probably offers the best option for COVID-19 control. Epitopes, mRNA, and *S* protein-RBD structure-based vaccines have been widely proposed and started [81,82]. Rapid reconstruction of SARS-CoV-2 using a synthetic genomics platform has been reported, and this technical advance is helpful for vaccine development [83]. Human ACE2 transgenic mouse and rhesus monkey models of COVID-19 have been well established for vaccine development [84], and someSARS-CoV-2 vaccines are already under clinical trial [85,86].

## 8. Conclusions and Perspective

In summary, SARS-CoV-2 is an emerging human CoV, and appears similar to previous SARS and MERS outbreaks. Bats are likely an important reservoir for SARS-CoV-2, and the current knowledge does not support the Huanan Seafood Market as the only source of infection. The main mode of transmission of SARS-CoV is through inhalation of respiratory droplets and indirect or direct contact, and infection has been estimated to have mean incubation period of 5.2 days and a R0 of 2.2. The most common factors behind COVID-19 mortality are older age and concomitant disease. The limited understanding about the pathogenesis of SARS-CoV-2 infection indicates that COVID-19 and SARS have similar pathogenesis, and also have differences such as massive mucus secretion in both lungs of critical patient. There are still no specific antiviral treatments or vaccines available. The most urgent task at the moment is to develop more interventions that will eventually allow the effective control of this arising severe viral infection.

The pandemic potential of human CoVs remains a great threat for global public health. However, human beings have not gained enough experience in previous battles with SARS and MERS. After the outbreak of COVID-19 in China, SARS-CoV-2 has received worldwide attention as an important pathogen in respiratory tract infection. Worldwide, over 330,000 people have fallen ill within four months. To lock down the spread of COVID-19, the Chinese government adopted an unprecedented containment strategy in Wuhan, the source of infection, and all other provinces or areas also declared an emergency response. However, in light of the current data, the ability of the virus to spread is beyond the current estimates. To provide a comprehensive summary to public health authorities and potential readers worldwide, we detailed the present understanding of COVID-19 and introduced the current state of development of measures in this review. The results of the present study are also valuable for informing further studies and health policies in preparation for COVID-19 outbreaks worldwide.
